# MSMEG_0918 is not Essential for the Growth of *Mycobacterium* *smegmatis*

**DOI:** 10.17912/micropub.biology.000891

**Published:** 2024-02-06

**Authors:** Eldana Bedru, Amala Bhagwat, Tanya Parish

**Affiliations:** 1 School of Medicine, University of Washington, Seattle, Washington, USA; 2 Center for Global Infectious Disease Research, Seattle Children’s Research Institute, Seattle, Washington, USA

## Abstract

Copper homeostasis plays a crucial role in mycobacteria. In *Mycobacterium tuberculosis* , Rv0474 is a copper-responsive regulator with a copper-binding motif but its homolog in *Mycobacterium smegmatis* , MSMEG_0918, lacks the copper-binding motif. We generated MSMEG_0918 knockdown strains of *M. smegmatis* using CRISPRi. We confirmed the strains had varying levels of *MSMEG_0918* expression using RT-PCR. We demonstrated that MSMEG_0918 under-expression did not alter the growth of *M. smegmatis* in standard aerobic culture as compared to the wild-type. Our knockdown strains (CHROME1 and CHROME2) could be further used towards understanding the role of MSMEG_0918 in *M. smegmatis* .

**Figure 1.**
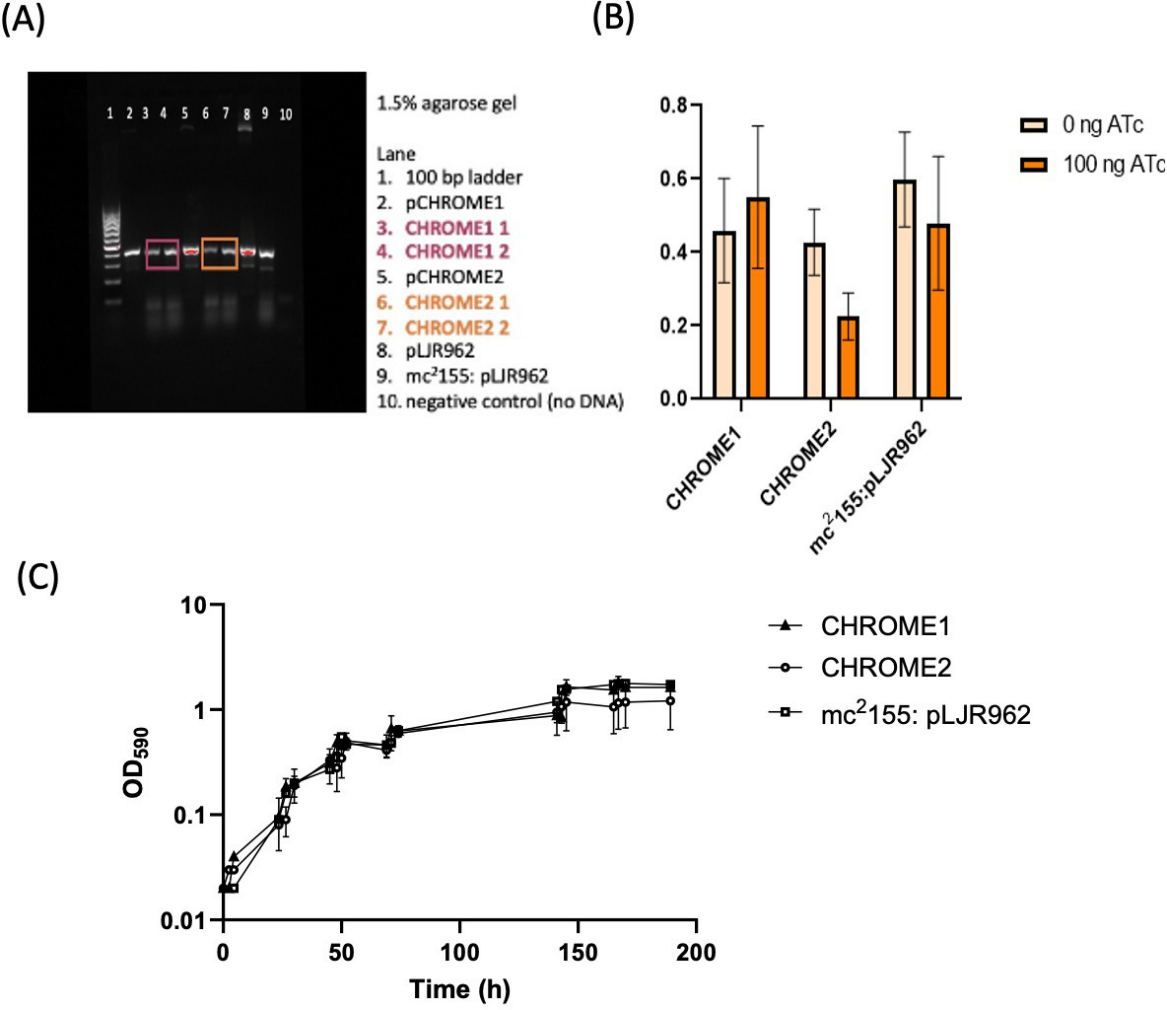
(A) Confirmation of the presence of pCHROME1 and pCHROME2 CRISPRi plasmids in recombinant strains of *M. smegmatis* . CHROME1 1 and CHROME1 2 are two independent transformants carrying plasmid pCHROME1. CHROME2 1 and CHROME2 2 are two independent transformants carrying plasmid pCHROME2. Plasmid presence was confirmed by colony PCR; plasmids pCHROME1 and pCHROME2 were used as positive controls; strain mc ^2^ 155:pLJR962 was used as the negative control. The expected product was 366 bp. (B) *MSMEG_0918* expression in recombinant strains was measured by RT-PCR and normalized against the reference gene *sigA* ( *MSMEG* _ *2758* ). Data were analyzed using a two-tailed t-test. *MSMEG_0918* expression was reduced by 2-fold in CHROME2 (p-value = 0.004) in the presence of 100 ng/mL ATc, indicative of an effective knockdown. (C) Growth of *M. smegmatis* strains in standard LB medium. Strains were inoculated at 1:10 dilution in 5 mL medium plus kanamycin selection (100 ng/mL ATc). CHROME1 = recombinant knockdown strain carrying pCHROME1. CHROME2 = recombinant knockdown strain carrying pCHROME2. mc ^2^ 155: pLJR962 = recombinant knockdown strain carrying empty plasmid pLJR962.

## Description

Tuberculosis (TB) is still one of the leading causes of death worldwide; the increase in multidrug-resistant *Mycobacterium tuberculosis* and lengthy drug treatment regimens, spanning >6 months, increase the urgency for new drugs (World Health Organization, 2022) . Efforts are being made to understand various mechanisms of resistance and to develop novel antimycobacterial drugs. Metal ion homeostasis plays a critical role in *M. tuberculosis* growth and its disruption could be seen as a drug development strategy (Neyrolles et al., 2015) . Copper is an important co-factor in several enzymes including those of the electron transport chain (Rowland & Niederweis, 2012) . While copper is critical for *M. tuberculosis* growth in vitro and inside macrophages (Neyrolles et al., 2015) , excess environmental copper levels are toxic and lead to cell death in *M. tuberculosis* (Rowland & Niederweis, 2012) . Copper poisoning is one of the primary mechanisms by which macrophages kill *M. tuberculosis* (Rowland & Niederweis, 2012) . In order to control intrabacterial copper levels, *M. tuberculosis* has an array of copper-responsive regulators, copper exporters and efflux pumps (Darwin, 2015) .

*M. tuberculosis* Rv0474 is a copper-responsive transcriptional regulator with two DNA binding domains and a copper binding motif (Raghunandanan et al., 2018) . Under low extracellular copper concentrations, Rv0474 represses its own expression (Raghunandanan et al., 2018) . At high concentrations of copper, Rv0474 autorepression is relieved, and Rv0474 can bind to the RNA polymerase promoter preventing its expression and leading to growth arrest. The Rv0474 homolog in the model organism, *Mycobacterium smegmatis mc ^2^ 155* is MSMEG_0918. Unlike Rv0474, MSMEG_0918 has one DNA binding motif and lacks the copper binding motif suggesting it plays a different role in this organism (Raghunandanan et al., 2018) . We were interested to determine if MSMEG_0918 was essential for *M. smegmatis* growth and its role. We therefore constructed *M. smegmatis* strains with reduced levels of expression using CRISPR interference.

We constructed plasmids carrying guide RNAs for MSMEG_0918; we designed two different oligo sets using the Pebble sgRNA design tool for mycobacteria (Wong & Rock, 2021) . The two plasmids were designed to have different levels of knockdown based on their PAM scores. In both plasmids expression of the CRISPR system is under the control of the tetracycline-inducible promoter. We electroporated both plasmids, and an empty vector into *M. smegmatis* mc ^2^ 155. We confirmed the presence of pCHROME1 and pCHROME2 by PCR using primers designed to amplify a small segment of the plasmid ( Figure 1A ). We determined the expression level of *MSMEG_0918* by RT-PCR in the recombinant strains. The level of expression was the same between strains in the absence of anhydrotetracycline (ATc) ( Figure 1B ). We expected a greater level of knockdown in the CHROME2 strain since the PAM score was higher and indeed, this was the only one of the two strains in which we saw a reduction of mRNA levels ( Figure 1B ) where we saw a 2-fold reduction in *MSMEG_0918* expression in the presence of 100 ng ATc.

We determined whether this level of expression had any effect on *M. smegmatis* growth ( Figure 1C ). Growth of CHROME1, CHROME2 and mc ^2^ 155:pLJR962 were monitored by OD590 ( Figure 1C ). Under-expression of MSMEG_0918 did not affect growth, as neither lag phase nor log phase were affected in the presence of ATc. While this is a negative phenotype, it leaves future possibilities to generate knockout of *MSMEG_0918* to establish its role in *M. smegmatis* .

## Methods

**Growth of *M. smegmatis***

*M. smegmatis* mc ^2^ 155 cultures were grown in Miller’s LB at 37°C. Recombinant strains mc ^2^ 155: pLJR962, CHROME1 and CHROME2 were supplemented with 20 μg/mL Kanamycin. To induce the CRISPRi system, 100 ng/mL anhydrotetracycline (ATc) was added.

**Design of sgRNA oligos for CRISPRi**

sgRNA oligos were constructed using the Pebble sgRNA design tool for mycobacteria (Wong & Rock, 2021) (Table 1). The lyophilized oligos were reconstituted to a concentration of 100 μM in molecular biology grade water and stored at -20°C.

**Generation of CRISPRi plasmids**

CRISPRi plasmids were generated by cloning the sgRNA oligos into the pLJR962 backbone. pLJR962 was a gift from Sarah Fortune (Addgene plasmid # 115162; http://n2t.net/addgene:115162 RRID:Addgene_115162). Generation of the CRISPRi plasmids were followed according to the protocol (Wong & Rock, 2021) as follows: sgRNA top and bottom oligos were annealed by adding 46 μL of oligo annealing buffer (50 mM Tris pH 7.5, 50 nM NaCl, 1 mM EDTA) to 2 μL each of 5 μM top and bottom oligos. The oligos were annealed in the Eppendorf Mastercycler thermocycler at 95°C for 2:00 and -0.1°C/sec to 25°C. pLJR962 was digested at the BsmBl site using 4 μL of New England BioLabs Inc. (NEB) BsmBl-v2 restriction enzyme (10,000 U/mL), 5 μL of NEBuffer 3.1, 2 μg (30.4 μL) of pLJR962 and 10.6 μL of nuclease free water. The reaction was incubated at 55°C for 4 hours. Afterwards, the digested plasmid was run against intact pLJR962 on a 0.8% agarose gel to check for the presence of a singular band. The annealed sgRNA oligos were cloned into the digested pLJR962 backbone through sticky end ligation. The ligation reaction consisted of 0.5 μL of annealed oligos, 0.3 μL of vector (BsmBl-digested), 0.5 μL of NEB 10X T4 DNA ligase buffer, 0.3 μL of NEB T4 DNA ligase (2,000,000 U/mL), and 3.4 μL of nuclease free water. Ligation was carried out at room temperature for 1.5 h and transformed into *Escherichia coli* . Single transformants were inoculated into 5 mL LB-Kanamycin and incubated overnight at 37°C with 100 rpm shaking. QIAprep Spin Miniprep Kit (Qiagen) was used to isolate plasmids; plasmids were sequenced using the oligo 5’-TTCCTGTGAAGAGCCATTGATAATG-3’ by GENEWIZ, Inc. Seattle, WA, USA.

**Electroporation**

Purified pCHROME1, pCHROME2 and empty pLJR962 backbone were electroporated into *M. smegmatis* mc ^2^ 155. Electroporation protocol was followed as described (Parish, 2021) as follows: *M. smegmatis* mc ^2^ 155 culture was incubated overnight at 37°C with 100 rpm shaking. Bacteria were incubated on ice for 1.5 h, washed three times with cold 10% w/v glycerol and resuspended in cold 10% w/v glycerol. Cells were stored in 0.2 mL aliquots at -80°C until use. Cells were thawed, harvested, and resuspended in 0.2 mL fresh 10% w/v glycerol. Aliquots were electroporated with ~2 μg of plasmid DNA using a 0.2 cm cuvette pulsed at 2.5 kV, 25 mF, 1000W. Cells were incubated on ice for 10 min, diluted into 5 mL LB and incubated at 37°C for 4 hours. Cells were harvested and plated onto LA with 20 μg/mL kanamycin selection. The plates were incubated at 37°C for 7 days; single transformants were streaked on LA with 20 μg/mL kanamycin.

**Colony PCR**

Primers FOR 5’- CCTCTGACCTGGGGATTTGC- 3’ and REV 5’- CGGCCTTTTTACGGTTCCTG- 3’ were used to confirm the presence of the plasmid. A loopful of transformed cells from LB-kanamycin agar plates was resuspended in 500 μL Tris- EDTA buffer and heated at 100°C for 10 min. Cells were removed by centrifugation and filtering through a 0.2 μm syringe filter. PCR reactions contained 10 μL of the DNA lysate, 1 μL of 10 μM CHROME forward primer, 1 μL of 10 μM CHROME reverse primer, 5 μL DMSO, 25 μL Quickload Taq 2X MasterMix, 8 μL nuclease-free water. The Eppendorf Mastercycler thermocycler was pre-heated to 95°C. The PCR settings used were: denaturation at 95˚C for 30 sec, annealing for 35 cycles at 95˚C for 15 sec, 55˚C for 30 sec, and 68˚C for 37 sec, and finally extension at 68˚C for 5 min. PCR products were run on a 1.5% agarose gel to look for a band of the expected product length of 366 bp.

**Growth Curves**

Recombinant strains were inoculated into 5 mL LB-kanamycin plus 100 ng/mL ATc (to induce the CRISPRi knockdown) and incubated at 37°C shaking overnight at 100 rpm. Overnight cultures were inoculated at 1/10 dilution into 5 mL LB-kanamycin medium in 20 mL Thermo Fisher Scientific Sterilin borosilicate glass culture tubes with screw caps. Cultures were incubated at 37°C and stirred magnetically. Growth was measured by monitoring OD _590_ over 8 days.

## Reagents

**Table 1. sgRNA oligo sequences**

**Table:** 

**Plasmid**	**Top Oligo**	**Bottom Oligo**	**PAM Site**	**PAM Score**
pCHROME1	5’-GGGAGTAGCCGGCTCCTCATCTGGTG-3’	5’-AAACCACCAGATGAGGAGCCGGCTAC-3’	5’-AGGGAAT-3’	47.3
pCHROME2	5’-GGGAGACCTCGCTGGGTTCGCTGGGTT-3’	5’-AAACAACCCAGCGAACCCAGCGAGGTC-3’	5’-CCAGGAT-3’	64.7
